# Direct effects of esmolol, ultra-short-acting β-blockers, on cardiac function, ion channels, and coronary arteries in guinea pigs

**DOI:** 10.1186/cc10401

**Published:** 2011-10-27

**Authors:** S Shibata

**Affiliations:** 1 Akita University Graduate School of Medicine, Department of Cell Physiology, Akita, Japan

## Introduction

β_1_-adrenergic antagonists have been recently used in septic patients to improve sepsis-induced immune, cardiovascular and coagulation dysfunction. But it is difficult and one is hesitant to use these drugs in septic shock patients who have already had hypotension because these drugs sometimes trigger excessive hypotension due to direct effects on heart function in addition to their β_1 _blocking effects. Since little is known about their acute direct effects on mammalian heart, we therefore evaluated the direct effects of esmolol, ultra-short-acting β-blockers, on cardiac performance and single cell-electrophysiology in guinea pig hearts, and compared these effects with those of landiolol.

## Methods

All animal experiments were approved by the University Animal Ethics Committee. Under deep anesthesia with pentobarbital, the heart was excised and mounted on a Langendorff apparatus to measure the coronary perfusion pressure (CPP). The saline-filled balloon was inserted into the left ventricle to measure the heart rate (HR) and systolic left ventricular pressure (sLVP). The coronary flow was maintained at a constant value during the experiments. Single ventricular cells were enzymatically isolated from hearts and cardiac ion currents were investigated by the patch clamp methods. Group comparisons were conducted by one-way repeated-measures analysis of variance with Dunnett's or Turkey's multiple comparison test. Differences at *P *< 0.05 were considered to denote significance.

## Results

Esmolol increased CPP in a concentration-dependent manner, and decreased both the sLVP and HR significantly at concentrations >10 μM. Esmolol also shortened the action potential duration (APD) in a concentration-dependent manner, and inhibited the inward rectifier K^+ ^current (I_K1_), while the L-type Ca^2+ ^current (I_CaL_) and outward current (I_Ks _and I_Kr_) and ATP-sensitive K^+ ^current were hardly affected. Furthermore, with the application of BAPTA from patch pipettes, the chelation of intracellular calcium ion did not antagonize APD shortening by esmolol. On the other hand, landiolol had minimal effects on cardiac coronary perfusion, cardiac contractility, action potential, and cardiac ionic currents. In the Kyoto Model computer simulation, sole inhibition of I_K1 _or I_CaL _failed to simulate APD shortening induced by esmolol.

## Conclusion

The present findings demonstrated that esmolol has more direct effects on the heart than landiolol; that is, the elevation of coronary perfusion pressure and negative inotropic action. The negative inotropic action is accompanied with the APD shortening in single cardiomyocytes. Inhibition of I_K1 _and I_CaL_, and inhibition of ionic current systems other than those we identified may be involved in the APD shortening caused by esmolol.

